# Simulating tumor microenvironment: changes in protein expression in an in vitro co-culture system

**DOI:** 10.1186/1475-2867-14-40

**Published:** 2014-05-13

**Authors:** Viviana Salvatore, Gabriella Teti, Silvia Bolzani, Stefano Focaroli, Sandra Durante, Maria Carla Mazzotti, Mirella Falconi

**Affiliations:** 1 Department for Biomedical and Neuromotor Sciences (DIBINEM), University of Bologna, via Irnerio 48, Bologna 40126, Italy

**Keywords:** Tumor microenvironment, Co-cultures, Cell-cell contacts, YKL-40, VEGF, MMP1, Osteosarcoma cell line, Human fibroblast cells

## Abstract

**Background:**

The role of the microenvironment during the initiation and progression of carcinogenesis is thought to be of critical importance, both for the enhanced understanding of fundamental cancer biology as well as for improving molecular diagnostics and therapeutics. The aim of this study was to establish an in vitro model based on a co-culture of healthy human fibroblasts (HFs) and human osteosarcoma cells (MG-63s) to simulate the microenvironment including tumor and healthy cells.

**Methods:**

The HFs and MG-63s were in vitro co-cultured for a period of time ranging from 24 h to 7 days. Cell morphology and organization were studied using phase contrast microscopy while the expression of Human Cartilage Glycoprotein 39 (YKL-40), Vascular Endothelial Growth Factor (VEGF) and Matrix Metalloprotease 1 (MMP1) was investigated by Real Time PCR and Western Blotting.

**Results:**

The results showed a characteristic disposition of tumor and healthy co-cultured cells in columns which are not visible in tumor and healthy cells grown singularly. The expression of YKL-40, VEGF and MMP1 significantly changed in co-cultured cells compared to HFs and MG-63s separately cultured.

**Conclusions:**

We concluded that the tumor microenvironment has an influence on the protein expression of the healthy surrounding tissues and the process of tumorigenicity.

## Background

In normal tissue, cells communicate through a complex network of interactions: physically, through direct contact or through the intervening extracellular matrix (ECM), and biochemically, through both soluble and insoluble signaling molecules [1]. Under persistent inflammatory conditions, the continual upregulation of enzymes, such as matrix metalloproteinases (MMPs) by stromal fibroblasts can disrupt the ECM; furthermore, invading immune cells can overproduce factors which promote abnormal gene expression [1]. These conditions are normally reversible but, when inflammation is sustained, the normal organization of cells might lead to gene expression instability within the healthy cells and the acquisition of tumorigenic potential [2]. These chronic inflammatory conditions help to establish a tumor microenvironment full of deranged proliferative signaling networks, which is largely orchestrated by inflammatory cells [3].

A tumor microenviroment includes invasive cancer cells and stromal cells, consisting in cancer-associated non-malignant fibroblasts, which provide an essential communication network via secretion of growth factors and chemokines. This condition leads to an altered ECM and provides additional oncogenic signals enhancing cancer-cell proliferation and invasion [4]. While the stromal cells are not malignant *per se*, their role in supporting cancer growth and development is vital to the survival of the tumor [5,6].

The aim of the study was to establish an in vitro model based on the co-culture of healthy human fibroblasts (HFs) and human osteosarcoma cells (MG-63s) to simulate the microenvironment of healthy tissue in connection with cancer cells. The final goal was to analyze the influence of microenvironment in both tumor and healthy cells, investigating the gene and protein expression of some biomarkers involved in cancer progression and invasion. A better understanding of the molecular mechanisms in the tumor microenvironment could be a crucial key to improving anti-cancer therapy.

During the past decade, there has been growing interest regarding glycoprotein YKL-40 [7,8], also known as chitinase 3–like 1 (CHI3L1) [9] and as human cartilage glycoprotein–39 (HC-gp39) [10]. YKL-40 is involved in angiogenesis [11] and the upregulation of the vascular endothelial growth factor expression (VEGF) in several cell lines [12-14]. Furthermore, YKL-40 plays a role in inflammation and tissue remodeling [15,16]. The aberrant and elevated expression of YKL-40 is largely associated with the pathogenesis of a variety of human diseases, such as rheumatoid- and osteoarthritis, hepatic fibrosis and asthma, which is connected to its pathological function and is associated with extracellular matrix remodeling, suggesting that the serum levels of YKL-40 serve as a diagnostic and prognostic biomarker [7].

Tumor invasion is also characterized by the increased ability of tumor cells to degrade ECM components through an enhanced expression of proteases, including matrix metalloproteinases secreted or anchored to the cell membrane. Among the membrane-type MMPs, matrix metalloproteinases 1 (MMP1) has been particularly implicated in pericellular proteolysis associated with cell migration and invasion [17]. An overexpression of MMP1 has been described in many types of human tumor tissues [18]. In addition, several studies have reported that MMP1 is produced by invasive tumor cells in vivo as well as in vitro and is thus associated with vascular remodeling, angiogenesis and tumor progression [17].

Acting together with metalloproteinases, the VEGF is a key regulator of physiological angiogenesis during embryogenesis, in vascular remodeling, wound healing, cell invasion, permeability, proliferation and survival [19]. The VEGF has also been implicated in pathological angiogenesis associated with tumors, intraocular neovascular disorders and other conditions. Inhibition of the VEGF is also being tested as a strategy for the prevention of angiogenesis and vascular leakage in several pathological conditions [20].

In the present study, we tested the expression of YKL-40, VEGF and MMP-1 in HFs and MG-63s seeded both alone and mixed in a co-culture system, for a period of time ranging from 24 h to 96 h. Light phase contrast microscopy was carried out to study the organization and orientation of cells in the co-culture from 4 to 7 days.

The influence of direct contact between the tumor cells and the non-malignant cells, and the interconnections of the biomarkers investigated and their role in tumor invasion and matrix remodeling were discussed.

## Results

### Morphological analysis by phase contrast microscopy

**Figure 1:**
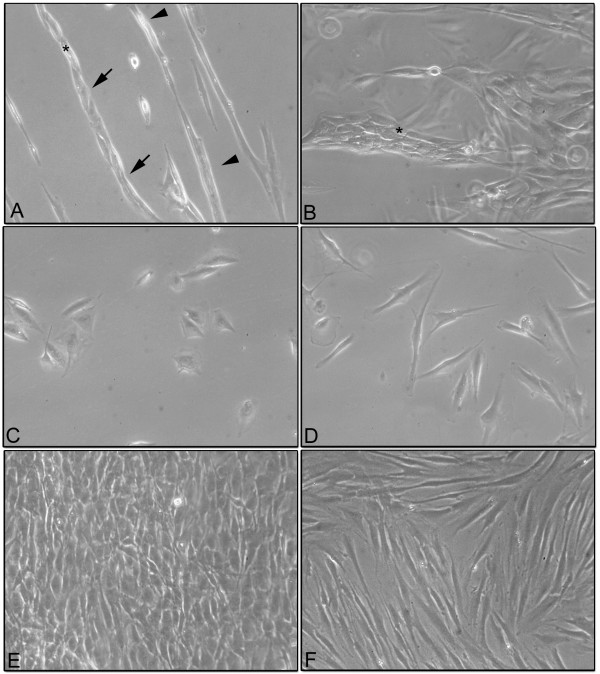
**Phase contrast microscopy. (A)** Co-culture of MG-63 cells and HFs grown for 4 days. Cord-like formations are well detected (*). HFs worked as a climbing frame on which the MG-63 cells (arrow) adhered and proliferated. Single HFs were also observed (arrowhead). **(B)** phase contrast microscopy of the co-culture of MG-63 cells and HFs grown for 7 days. Thicker cord-like formations were detected (*). **(C)** phase contrast microscopy of the co-culture of MG-63 cells grown separately in monolayers for 24 h. **(D)** phase contrast microscopy of HFs grown in monolayers for 24 h. **(E)** phase contrast microscopy of MG-63 cells grown in monolayers for 7 days. **(F)** phase contrast microscopy of HFs grown in monolayers for 7 days. Magnification for all the images was 100X. Aperture number is 0.25.

After 4 days of co-culture, the MG-63s and the HFs showed a specific and characteristic organization based on cord-like formations (Figure 1A). After 7 days of co-culture, the cord-like formations were larger and the HFs were almost completely covered by MG-63 cells (Figure 1B).

On the contrary, control samples of the MG-63s and the HFs, grown separately, showed their characteristic cell morphology. The MG-63s had a polygonal morphology after 24 h of culture (Figure 1C) while the HFs showed a fibroblastic morphology with an elongated cell body after 24 h of culture (Figure 1D). At the end of 7 days of culture, both HFs and MG-63s had reached confluence (Figure 1E and F).

### qRT-PCR of YKL-40 and VEGF in co-cultured HFs and MG-63 cells

To examine changes in protein expression during the coexistence of HFs and MG-63 cells, the mRNA expression of YKL-40 and VEGF was evaluated using a quantitative real time polymerase chain reaction (qRT-PCR) in cells grown in co-culture, and the results were compared to the same cells grown separately as controls.

**Figure 2:**
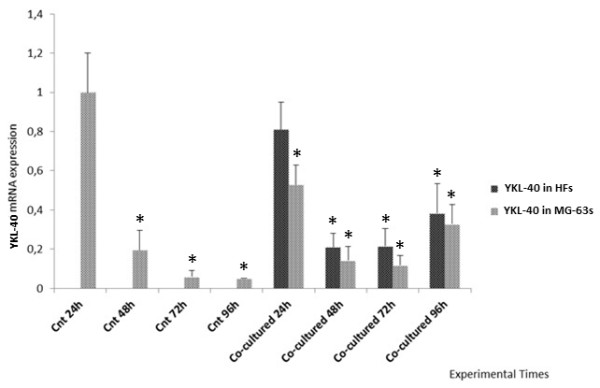
**Real Time PCR for YKL-40.** Expression of HFs and MG-63 cells independently grown and co-cultured. *represents a significant difference from the MG-63 control cells, P < 0.05.

The expression of YKL-40 in HF cells seeded separately and used as controls was almost absent. Indeed, YKL-40 expression was the highest at 24 h; it subsequently decreased and remained constant up to 96 h (Figure 2). In MG-63 cells, the expression of YKL-40 was very high at 24 h in controls seeded separately and then decreased until 96 h. In the MG-63 cells grown in co-culture, a similar trend to the HFs grown in co-culture was observed, but with a slightly lower intensity of expression (Figure 2).

**Figure 3:**
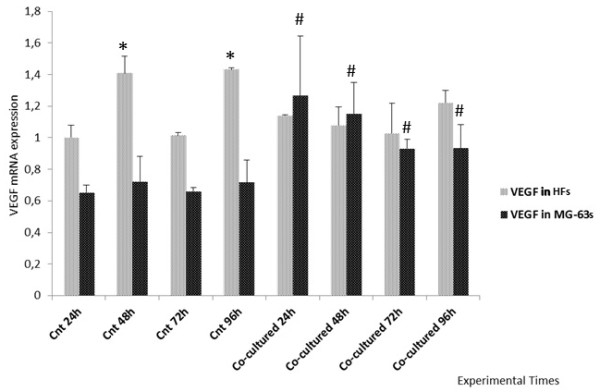
**Real Time PCR for VEGF.** Expression of HFs and MG-63 cells independently grown and co-cultured. *represents a significant difference from the HF control sample at 24 h, P < 0.05. # represents a significant difference from the MG-63 control cells at 24 h, P < 0.05.

The VEGF showed constant expression in the HFs both grown in co-culture and separately while, in MG-63 cells, it was higher in co-culture with respect to the control cells seeded separately (Figure 3).

### Western Blotting and densitometric analysis

**Figure 4:**
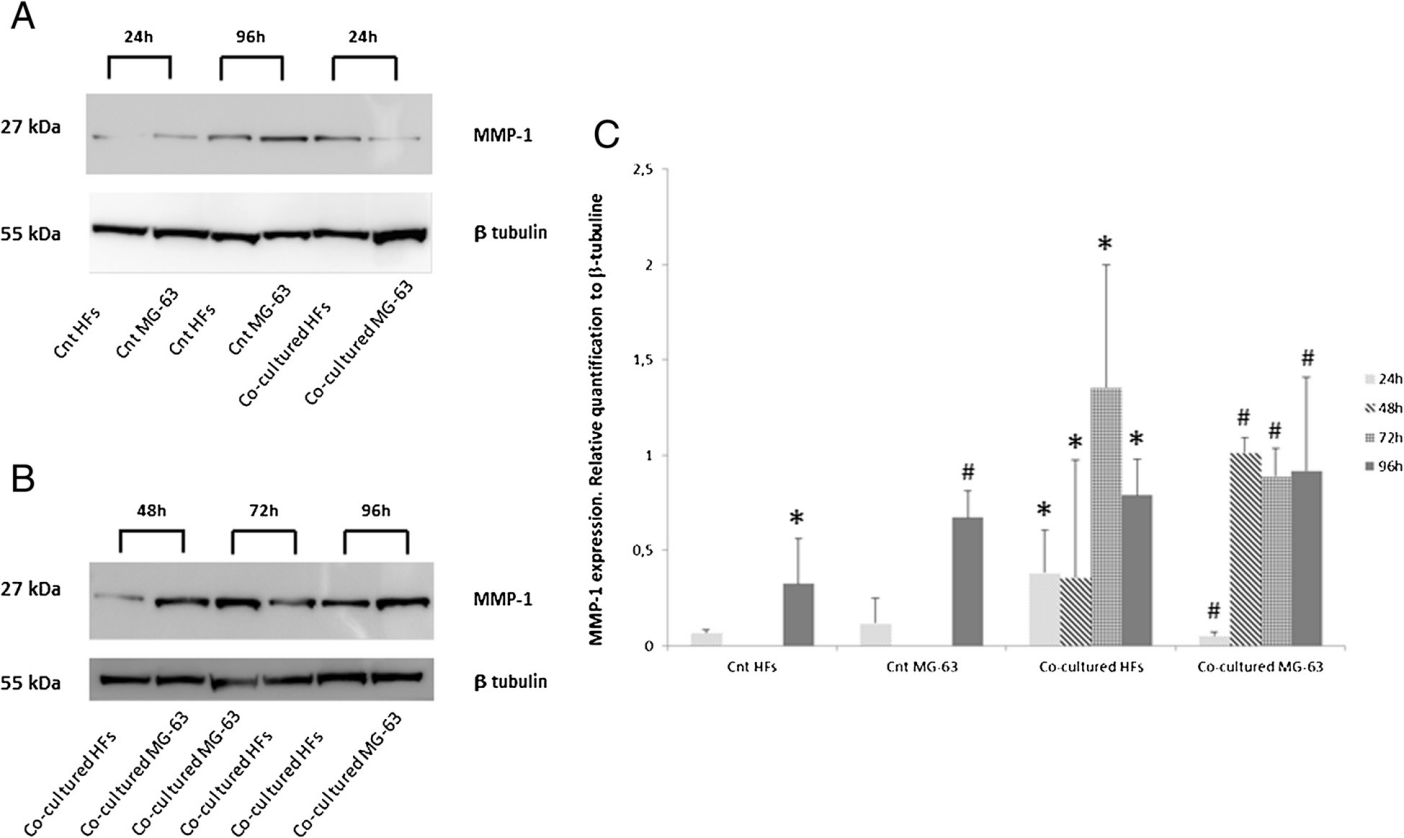
**Western Blot for MMP1. (A)** MMP1 expression of HFs and MG-63 cells independently grown and **(B)** co-cultured. **(C)** Densitometric analysis of MMP1 expression relative to HFs and MG-63 cells independently grown and co-cultured. *represents a significant difference from the HF control sample, P < 0.05. # represents a significant difference from the MG-63 control cells, P < 0.05.

Control HFs showed a low level of MMP1 expression after 24 h of culture while it increased after 96 h. On the contrary, MG-63 control cells demonstrated a higher expression of MMP1 protein after 24 h and 96 h of culture as compared to HFs (Figure 4A).

In the co-cultured cells, the intensity of the MMP1 bands increased considerably over time, reaching the highest intensity in HFs at 72 h and in MG-63s at 48 h (Figures 4A and B), as demonstrated by densitometric analysis (Figure 4C).

## Discussion

For many years, laboratories have sought to understand the mechanisms by which cells respond to their microenvironment, and how these signals are integrated to specify programs of gene expression and, ultimately, tissue phenotype. Understanding these processes and their alteration will engender a greater appreciation of the mechanisms involved in tumor progression.

The scientific literature has shown how remodeling the ECM and producing aberrations can stimulate the development of cancer [21].

The aim of the present study was to develop an in vitro methodology for investigating the interactions between non-malignant cells and tumor cells, and to verify how the microenvironment influences cells in the co-culture system.

During malignant transformation, healthy and tumor cells are increasingly described as networks of their physical and signaling contacts [22]; with this network analysis approach, we chose to co-culture a healthy and non-malignant population of human fibroblast cells with a human osteosarcoma cell line, MG-63s, to study the morphological and molecular changes originated by this direct interaction.

The morphological analysis of cells independently co-cultured in monolayers demonstrated a well-defined and different cell morphology of MG-63s and HFs in contrast to monolayers of co-cultured HFs and MG-63s which showed cord-like structures after 4 days of culture, thickening after 7 days. We suggested that, in cord-like structures, the fibroblasts worked as a climbing frame on which the MG-63 cells adhered and proliferated. The absence of this type of organization in MG-63 cells and HFs grown separately suggests a morphological rearrangement due to modifications in the microenvironment of the co-culture system. Fauquette and colleagues [23] demonstrated that the cord-like morphogenesis was supported by changes in the cell surface receptor after contact between tumor cells and healthy cells, which was eventually associated with an elevated expression of adhesive molecules, leading to cell contact reorganization and cell-matrix changes. These data correlated well with our results and with tumor invasiveness ability.

To verify whether the microenvironment has an influence on cells grown in co-culture, the expression of the YKL-40, VEGF and MMP1 proteins involved in the progression of pathological conditions was analyzed. Some years ago, Johansen et al. [24] identified a protein called YKL-40, secreted in vitro in several types of solid tumors and in large amounts by MG-63 cells. On the contrary, there are no data regarding in vitro YLK-40 expression in HFs [16]. For this reason, we have chosen the YKL-40 protein as the main marker for verifying the influence of tumor cells grown in contact with healthy cells.

As reported in the literature [25], YKL-40 was identified as a tumor angiogenesis factor, inducing coordination of membrane-bound receptor syndecan 1 and integrin αvβ3, and activating an intracellular signaling cascade including FAK (focal adhesion kinase), Erk 1, and Erk 2 [26]. Furthermore, YKL-40 not only increased the expression of Flk-1/KDR (phosphatidylinositol 3-kinase/akt signal), VEGF receptor 2 which mediates VEGF angiogenesis [27], but also activated the tyrosine phosphorylated form of Flk-1/KDR, possibly leading to a synergistic effect on angiogenic signaling activation. YKL-40 purified from the MG-63 osteosarcoma cell line has growth factor activity for fibroblast cell lines [15]. The YKL-40 secreted by cancer cells has a role in the mutation of the fibroblasts surrounding the tumor, including the activation of fibroblast morphologic transformation, secretion of MMPs and neovascularization. Therefore, YLK-40 promotes the proliferation, differentiation and invasion of cancer cells and the destruction of stroma [28-30]. The overexpression of MMP1 has been shown in tumor tissues and has been suggested to be associated with tumor invasion and metastasis [18]. The production of MMP1 is stimulated in fibroblasts by growth factors and cytokines; it is postulated that YKL-40 acts synergistically as a growth factor with VEGF, stimulating MMP1 expression [13,19].

Starting from these previous data, we investigated the expression of YKL-40, the VEGF and MMP1 using Real Time PCR and Western Blotting, first in cells grown separately and then in the co-culture system, in order to compare the protein expression trend in both cellular groups. The final goal was to verify whether HFs underwent changes after direct contact with tumoral cells.

Real Time results demonstrated that YKL-40 was expressed only in MG-63 control cells, in accordance with the literature [16], and was not expressed in HF control cells, during the entire experimental time. In addition, our data gave an unexpected result; in the co-culture system, we found the expression of YKL-40 even in HFs, although fibroblasts do not normally express this protein in vitro. The HFs co-cultured with MG-63s effectively showed an elevated expression of YKL-40 after 24 h, and then remained almost constant during the experiment.

The expression of the VEGF is quite constant in the HFs used as controls and in co-cultures, but increases in MG-63 cells when co-cultured. We suggest that the tumor cell-conditioned medium exhibited an impact on YKL-40 expression in HFs, and on VEGF expression in MG-63 cells, perhaps due to the growth factor activity of YKL-40 secreted in the tumor cell-conditioned medium and to its direct role in stimulating VEGF expression and ECM remodeling.

Western Blot analyses showed weak MMP1 expression in control HFs and a more elevated expression in control MG-63 cells. In both samples, the level of MMP1 gradually increased during the time of culture. Co-cultures of HFs and MG-63 cells showed high levels of MMP1 after only 24 h and they greatly increased up to 96 h, suggesting a strong influence of the tumor microenviroment on the upregulation of the matrix metalloproteinases responsible for the disruption of the ECM and tumor invasiveness [2]. In consideration of our results, we could better postulate the role of YKL-40 in the tumor microenvironment.

Ngrnyuang et al. have recently reported [14] that the effects of YKL-40 are cell type-dependent, probably due to the different biochemical composition between cell lines. Human fibroblasts and MG-63s represent two biologically different cellular types involved in parallel roles during tumor progression. Tumor cells, releasing pro-inflammatory factors, are able to induce the activity of NF-kB (Nuclear Factor kappa B) in fibroblast cells which, in turn, induce the release of growth factors and cytokines in the adjacent extracellular matrix, enhancing an inflammatory microenvironment and promoting changes in the ECM. Our hypothesis is that YKL-40 may have synergistic effects; on HFs, it can act similarly to insulin-like growth factor-1, leading to an inflammatory condition and promoting an up-regulation in MMP-1 expression, in order to remodel the extracellular matrix. In addition to the inflammatory conditions, the expression of YKL-40 in MG-63s, correlated with those of the VEGF, suggests that YKL-40 acts as an angiogenic factor in tumor cells, as has been reported in the literature.

Following these preliminary data, it could be of great interest to examine the effect of the siRNA knockdown of YKL-40 in MG-63 cells on the changes reported in protein expression in the co-cultures. These experiments are currently in progress.

## Conclusions

In conclusion, this study showed that cancer evolution can involve changes in the microenvironment as a result of oncogenic stress within healthy cells. The phenotypic reorganization between fibroblasts and MG-63 cells, extracellular matrix remodeling, changes in YLK-40 protein expression and increased VEGF expression are indications of cancer progression.

Our experimental approach suggested a realistic in vitro model of the tumor microenvironment, useful for better investigating the complex mechanisms which transform a healthy tissue environment to a malignant state.

## Methods

### Primary culture of HFs

Human dental ligament (HDL) tissues were collected from the mid-third of roots of teeth extracted for orthodontic reasons, following informed consent of the patients. After several washes in PBS, the HDL tissues were cut into small pieces and placed into culture dishes with 1 mL of Dulbecco’s Modified Essential Medium (DMEM) (Invitrogen, Carlsbad, CA, USA) supplemented with 10% (v/v) fetal bovine serum (FBS), penicillin (100 mg/mL) and streptomycin (10 mg/mL). The culture medium was changed twice a week. When the HFs were subconfluent (70–80%) they were scraped off using 0.05% trypsin/EDTA (Gibco, Grand Island, NE), washed and placed into T75 flasks. The cells obtained were cultured at 37°C in a humidified atmosphere of 5% CO_2_. Cells from passages 3 to 10 were utilized for the following experiments.

### MG-63 cell culture

The MG-63 cell line was purchased from ATCC® CRL-1427™ (USA) and cultivated in DMEM (Invitrogen, Carlsbad, CA, USA) containing 10% FBS, supplemented with 10% (v/v) FBS, penicillin (100 mg/mL) and streptomycin (10 mg/mL) according to the recommendation of the supplier. The cells were cultured in T25 flasks (Nunc, USA) in a humidified incubator at 37°C in a 5% CO_2_ humidified atmosphere. For passaging, the cells were detached with trypsin/EDTA and subsequently replated.

### Co-cultures of HFs and MG-63 cells

The HFs and the MG-63 cells were seeded at the same density (15 × 103 cells/mL each) in T75 flasks and cultured in DMEM F-12 supplemented with 10% (v/v) FBS, penicillin (100 mg/mL) and streptomycin (10 mg/mL) for 24 h, 48 h, 72 h and 96 h.

As controls, monocultures of MG-63 cells and HFs were seeded separately in T75 flasks and were cultured under the same conditions as the co-cultures. The cell medium was replaced twice a week.

### Monostrate cultures and morphological analysis using phase contrast microscopy

Ten ×10^3^ cells/ml were seeded on glass slides in 6 multiwells and co-cultured for 4 and 7 days. At the end of each experimental time point, the samples were observed using a phase contrast microscope Motic AE21. The images were recorded using Visicam 3.0 and analyzed by VisiCam Image Analyzer software, version 6.1.3.3.

The images of Figure 1 were representative for three independent experiments.

### Cell separation

At every experimental time point, the cells seeded in co-cultures were detached, collected and counted for separation with MS Columns and Anti-Fibroblast Microbeads System (MACS, Miltenyi Biotec) according to the manufacturer’s instructions. Briefly, the cells were centrifuged, and the pellets were suspended in MACS buffer solution. The HFs were then magnetically labeled with human Anti-Fibroblast MicroBeads while the unlabeled MG-63s were run through the column after 3 washes with MACS buffer. By so doing, the MG-63 fraction was depleted of HFs. After removing the column from the magnetic field, the magnetically retained HFs were eluted as the positively-selected cell fraction. The separation efficiency guaranteed by the MACS Miltenyi System was more than 90%.

### RNA extraction

Total RNA from control and co-cultured pellets was extracted using Nucleospin RNA II (Macherey-Nagel) and quantified using a NanoDrop® ND-1000 UV–vis Spectrophotometer (Thermo Scientific, Wilmington, DE, USA). One μg of total RNA was reverse transcribed using a high capacity cDNA Reverse Transcription kit (Applied Biosystem, Life Technologies, Monza Italy) according to the manufacturer’s instructions.

### Real time PCR

The expression of mRNA was analyzed by quantitative Real Time PCR using 7500 Real Time PCR (Applied Biosystem, Life Technologies, Monza, Italy). All reactions were carried out in a 25 μL reaction volume in triplicate. For the analysis, the following TaqMan assays (Applied Biosystems, Life Technologies, Monza, Italy) were used: Hs00609691_m1 for YKL-40 and Hs00900055_m1 for VGEF. All samples were normalized to glyceraldehyde 3-phosphate dehydrogenase (GAPDH, Hs99999905_m1) expression.

The data were representative of three independent experiments and showed the average of triplicates ± SD.

### Western blot analysis and densitometric analysis

At each experimental time point, the cell pellets were lysed for 30 minutes using a RIPA extraction buffer (Invitrogen, Life Technologies, Monza Italy) supplemented with a protease inhibitor cocktail (Sigma Aldrich, St Louis, Missouri, USA), 1 mM PMSF and 0.15% β-mercaptoethanol (Fluka, Sigma Aldrick, St Louis, Missouri, USA). The samples were centrifuged at 14,000 rpm for 10 minutes at 4°C, and the total protein amounts were assayed using Bradford reagent (Sigma Aldrich, St. Louis, Missouri, USA).

Twenty μg of total protein were resolved on NuPAGE® SDS-PAGE pre-cast gels (4-12%) (Invitrogen, Life Technologies, Monza, Italy), and the protein was transferred to a nitrocellulose membrane (GE Healthcare Europe GmbH, Milan, Italy), blocked with no fat dry milk (Sigma Aldrich, St. Louis, Missouri, USA) for 30 min at room temperature (RT), and immunolabeled with anti-MMP1 1:500 in TBS pH7.5 and anti-β tubulin 1:10000 in TBS pH7.5 overnight at 4°C. The bands were visualized using an ECL Advanced TM Western blotting detection kit (GE Healthcare Europe GmbH, Milan, Italy) and the images were recorded with a Kodak digital image station (Eastman Kodak, Rochester, NY, USA).

Band densitometry was measured using Image J software (National Institutes of Health) and the intensities of the specific protein bands were corrected for equal β-tubulin loading; they were expressed as relative to the intensity of the control sample. The relative quantitation of the western blots was expressed relative to the β-tubulin present on each blot.

The images of Figure 4 were representative of three independent experiments. The densitometry data were representative of three independent experiments and showed the average of triplicates ± SD.

### Statistical analysis

The statistical analysis was carried out using GRAPH PAD PRISM 5.0 software (San Diego, CA) by applying ANOVA and the Dunnet’s multiple comparison test. The differences were considered significant at p < 0.05.

## Abbreviations

ECM: Extracellular matrix; FAK: Focal adhesion kinase; HDL: Human dental ligament; HFs: Human fibroblasts; MMP1: Matrix metalloprotease 1; MMPs: Matrix metalloproteinases; VEGF: Vascular Endothelial Growth Factor; YKL-40: Human cartilage glycoprotein–39 (N-term. Tyrosine (Y), Lysine (K) and Leucine (L) molecular mass 40 kDa (40).

## Competing interests

The authors declare no financial interest or sources of research funding which could affect integrity of the scientific work presented.

## Authors’ contributions

VS, GT and MF participated in the design of the study and in the analysis of the data. VS and SB carried out the experimental work. VS and GT carried out the statistical analysis. The acquisition of data was carried out by SF, SD and MCM. They also helped to draft the manuscript. MF has given final approval of the version to be published. All the authors have read and approved the final manuscript.
